# Plantamajoside from *Plantago asiatica* modulates human umbilical vein endothelial cell dysfunction by glyceraldehyde-induced AGEs via MAPK/NF-κB

**DOI:** 10.1186/s12906-017-1570-1

**Published:** 2017-01-21

**Authors:** Won-rak Son, Mi-Hyun Nam, Chung-Oui Hong, Yoonsook Kim, Kwang-Won Lee

**Affiliations:** 1 Department of Biotechnology, College of Life Science and Biotechnology, Korea University, Seoul, 02841 South Korea; 2 Department of Ophthalmology, University of Colorado School of Medicine, Aurora, CO 80045 USA; 3 International Ginseng & Herb Research Institute, Geumsan-gu, Chungnam 32724 Republic of Korea; 4 Korea Food Research Institute, Seongnam-si, Gyeonggi 13539 South Korea

**Keywords:** Endothelial dysfunction, Adhesion molecule, Monocyte adhesion, Advanced glycation end-products, Plantamajoside

## Abstract

**Background:**

*Plantago asiatica* has been traditionally used for traditional medicine around East Asia. Plantamajoside (PM), which is isolated from this plant, is known for biological properties including anti-inflammation and antioxidant activity. To demonstrate the biological activity of PM against endothelial dysfunction induced by advanced glycation end-products (AGEs), a cellular inflammatory mechanism system was evaluated in human umbilical vein endothelial cells (HUVECs).

**Methods:**

We obtained PM through previous research in our laboratory. We formed the AGEs from bovine serum albumin with glyceraldehyde in the dark for seven days. To confirm the modulation of the inflammatory mechanism in endothelial dysfunction, we quantified the various pro-inflammatory cytokines and endothelial dysfunction-related proteins in the HUVECs with Western blotting and with real-time and quantitative real-time polymerase chain reactions.

**Results:**

Co-treatment with PM and AGEs significantly suppressed inflammatory cytokines and adhesion molecule expression. Moreover, the PM treatment for down-regulated inflammatory signals and blocked monocyte adhesion on the HUVECs.

**Conclusions:**

Theses results demonstrated that PM, as a potential natural compound, protects AGE-induced endothelial cells against inflammatory cellular dysfunction.

**Electronic supplementary material:**

The online version of this article (doi:10.1186/s12906-017-1570-1) contains supplementary material, which is available to authorized users.

## Background

Arterial lesions are mediated by complicated manifestation between vascular endothelial dysfunction and proliferation in vascular smooth muscle cells [1, 2]. Atherosclerosis is a critical inflammatory arterial disease and is one of the most fatal complications of diabetes, initiated by reactive oxygen species (ROS) induced by the formation of advanced glycation end-products (AGEs) [3]. During hyperglycemia, AGEs are formed by non-enzymatic reaction with aldehydes and free amino sites and are considered a risk factor [4]. Excessive formation of intracellular ROS in response to AGEs is reported as a crucial mediator in the development of vascular lesions as well as diabetic cardiomyopathy, nephropathy, retinopathy, and peripheral neurological damage [5–8]. The interaction between AGEs and RAGE (an AGE receptor) activates NAD(P)H oxidase and mitochondria, which generates ROS and induces inflammatory cytokines through multiple signal cascades [9–12]. At the same time as the AGE-RAGE interaction, mitogen-activated protein kinases (MAPKs) including extracellular signal-regulated kinases 1/2 (ERK 1/2), p38 and c-jun N-terminal kinases (JNK) are transduced, resulting in transcription factor activation and the expression of adhesion molecules [13–17]. As a pre-dispositional factor in atherosclerosis, leukocyte-endothelial adhesion is known to initiate endothelial dysfunction, followed by increased expressions of pro-inflammatory cytokines including tumor necrosis factor alpha (TNF-α), interleukin-6 (IL-6) and monocyte chemoattractant protein-1 (MCP-1) [18–20]. Moreover, monocytes transmigrate to the endothelium through molecules such as intercellular adhesion molecules-1 (ICAM-1) and vascular cell adhesion molecules-1 (VCAM-1), and they are ultimately converted to the activated form, known as M1-like macrophages [21].

In various inflammation-related diseases, nuclear factor-kappaB (NF-κB) transcription factor is highly activated, and it is known as a pivotal inducer of pro-inflammatory cytokines, chemokines and adhesion molecules [22–25]. Regulating NF-κB has been considered an important check-point to overcome inflammatory diseases in recent studies. NF-κB complex is composed of I-kappa B (IκB) and heterodimer, which is composed of p50 and p65 subunits in cytoplasm. Upon activation of the NF-κB complex, IκB is released from the complex, phosphorylated, and then degraded by proteasomes. At the same time, p50 and p65 subunits are translocated to nuclei and bind to transcription sites [26, 27].

*Plantago asiatica* is traditionally used as a natural plant medicine in East Asia, and it has been reported to have biological activity including antipyretic, wound healing, anti-cancer, anti-virus and anti-hepatitis properties [28, 29]. Plantamajoside (PM), a phenylethanoid glycoside compound from *P. asiatica*, has been reported to inhibit glycation activity [30] and to have anti-inflammation and antioxidant properties [31, 32] as well as nephroprotective effects against heavy metals in an in vivo model [33]; however, the molecular mechanism of how PM modulates endothelial dysfunction remains uncertain. Therefore, in our study, we investigated the preventive effects of PM on endothelium dysfunction mediated by glyceraldehyde-induced AGEs (glycer-AGEs) using human umbilical vein endothelial cells.

## Methods

### Plant material and preparing the PM

We obtained the *P. asiatica* from a traditional market (Kyungdong Herb Market, Seoul, Korea). The PM, which was extracted from *P. asiatica*, was isolated and identified by Professor B. W. Kang (College of Life Sciences & Biotechnology, Korea University) and obtained through previous research in our laboratory. We deposited the voucher specimens at the Herbarium of Korea University (Register number, H-212).

Briefly, the plant powder was immersed in 100% methanol (MeOH) within a 100 mL/g ratio at 70 °C for 3 h in a reflux condenser. After the dissoluble materials were filtered, the combined filtrate was concentrated with rotary vacuum evaporation and lyophilized for the dried residue. To fractionate the dried *P. asiatica* extract, the dried residue was suspended in H_2_O and then sequentially fractionated with *n-*hexane, chloroform, ethyl acetate (EtOAc) and *n-*butanol (BuOH). The BuOH-soluble portion was applied to a 60 g silica gel 60 column (63–200 μm, Merck) and eluted with gradient amounts of MeOH in EtOAc. The active fraction of EtOAc and MeOH (90:10, v/v) was purified by eluting in MeOH with a Sephadex TM LH-20 column (Amersham Biosciences, Uppsala, Sweden), followed by PR-μ-BondaPak C18 column chromatography (Waters, Milford, MA, USA) for the active compound [30].

### Chemicals and materials

We obtained endothelial cell basal medium-2 (EGM-2) from Lonza Cambrex (Nottingham, UK) and obtained M-199 and RPMI 1640 tissue culture mediums from GIBCO (Grand Island, NY, USA). We obtained the anti-ICAM-1, anti-RAGE, anti-ERK, anti-phosphorylated-ERK, anti-JNK, anti-phosphorylated-JNK, anti-phosphorylated-p38, anti-NF-κB and anti-PCNA antibodies from Santa Cruz Biotechnology, Inc. (Heidelberg, Germany) and the antibodies against anti-VCAM-1, anti-p38, and phosphorylated-IkappaBα, PD98059 (an ERK1/2 inhibitor), SP600125 (a JNK inhibitor), and SB203580 (a p38 inhibitor) from Cell Signaling Technology Inc. (Danvers, MA, USA). We obtained the anti-GAPDH antibody from Merck (Darmstadt, Germany), and all the other most highly purified chemicals were commercially provided from Sigma-Aldrich (St. Louis, MO, USA).

### Preparing the glyceraldehyde-induced AGEs

Bovine serum albumin (BSA, 20 mg/mL) was incubated with 20 mM glyceraldehyde in 0.1 M potassium phosphate buffer saline (PBS, pH 7.4) that contained 1 mM diethylene triamine pentaacetic acid for 7d at 37 °C in the dark. AGE formation was confirmed using fluorescence spectroscopy (excitation 370 nm/emission 440 nm), and we noted an approximately 200-fold increase in glycer-AGEs compared with the control BSA (Additional file 1: Figure S1), which strongly suggested that AGEs were formed. After 7d, reduced BSA (rBSA) was recombined with 80 mM sodium borohydride for 30 min to delete the unexpected glycated products during incubation. After the unreacted sugar or small molecular reactants in the 0.1 M PBS were dialyzed, the prepared glycer-AGEs, and rBSA were sterilized by filtration and stored at −20 °C.

### Cell culture and treatment

Primary human umbilical vein endothelial cells (HUVECs) were purchased and maintained with EGM-2 medium that contained endothelial growth supplements (Lonza, Seoul, Korea) with 2% fetal bovine serum (FBS) at 37 °C in a retained 5% CO_2_ incubator. The human leukemia THP-1 cells were cultured in an RPMI 1640 medium with 0.05 mM 2-mercaptoethanol with 10% inactivated FBS. To treat with the glycer-AGEs, the HUVECs were co-treated with different concentrations of PM and 100 μg/mL of AGEs using FBS-free media. In specific inhibitor-treated experiments, the inhibitors were pre-treated for 1 h and removed before the sample treatments.

### Measuring cell cytotoxicity

We measured the cell cytotoxicity of PM or *N*-acetylcysteine (NAC) in the endothelial cells using colorimetric MTT assay. We seeded wells at 1 × 10^4^ cells/well and grew the cells in 96-well culture plates. After 24 h, we applied differing concentrations of PM or NAC with 100 μg/mL of AGEs for the indicated times. After the treatments, we removed the supernatants and incubated the cells with 48 μL of MTT reagent (5 mg/mL) for 4 h. We quantified the reduction of MTT to formazan, which is dissolved by DMSO, at 540 nm using a multi-plate reader (Bio-Tek Instruments, Winooski, VT, USA) and assessed cell viability as the percentage of untreated control cells.

### Determining the intracellular ROS in the HUVECs

The HUVECs (1 × 10^4^ cells/well) were cultured in 96-well culture plates, and 10 μM DCF-DA were pre-incubated in a culture medium at 37 °C in a CO_2_ incubator for 30 min. After they were washed with PBS, the cells were co-treated with differing concentrations of PM with 100 μg/mL of glycer-AGEs for 3 h. For specific inhibitors, 10 μM rotenone, 10 μM apocynin and 10 μ Mallopurinol were pre-treated for 1 h before sample treatments. We analyzed the oxidation of DCF-DA to DCF by intracellular ROS by measuring the fluorescence at excitation of 485 nm and emission of 535 nm using a fluorescence spectrophotometer (VICTOR3^TM^, PerkinElmer, Waltham, MA, USA). Intracellular ROS levels are expressed as percentages of rBSA-treated cells.

### Cell extraction and Western blotting

We cultured the HUVECs (1 × 10^6^ cells) in 60-mm dishes and co-incubated them with or without 10 μM PM or 1000 μM NAC in the presence of 100 μg/mL of AGEs for the indicated times of 60 min to 24 h. We pre-treated the specific inhibitors for 1 h before the sample co-treatments, washing them twice with cold PBS, suspending them in RIPA lysis buffer (ELPIS Biotech Inc., Daejeon, Korea) for total cell lysates with protease inhibitors (5 μg/mLof leupeptin, 5 μg/mLaprotinin and 1 mM PMSF), and then centrifuging them at 13,000 × *g* for 20 min at 4 °C. For cell lysates with phosphorylated proteins, we added phosphatase inhibitor cocktail 2 (Sigma-Aldrich, St. Louis, MO, USA) to the same lysis buffer as before. To determine nuclear fractionation, we prepared the cells with PBS and cytoplasmic fraction separated with a cytosol extract buffer (10 mM Hepes, pH 7.8 with 10 mM KCl, 0.1 mM EDTA, 1 mM DTT and 10% NP-40). We prepared the nuclear extract with a nuclear extraction buffer (50 mM Hepes, pH 7.8 with 50 mM KCl, 300 mM NaCl, 0.1 mM EDTA, 1 mM DTT and 20% glycerol) after removing the cytosolic extracts and strongly vortexing the cells for 10 min at 4 °C. We then determined the protein contents in the total and nuclear fractions using a BCA protein assay (Pierce Biotechnology, Waltham, MA, USA). We reconstituted the samples in a loading buffer that contained 60 mM Tris–HCl, pH 6.8, 10% glycerol, 2% sodium dodecyl sulfate (SDS), 1% β-mercaptoethanol and 0.02% bromophenol blue, and boiled the mixture for 10 min at 100 °C. We loaded equal amounts of the denaturalized proteins into each lane, separated them by 10% SDS-polyacrylamide gel electrophoresis, and transferred them to PVDF membranes (Merck Millipore, Billerica, MA, USA). We blocked the transferred membranes in 5% non-fat dried milk in Tris-buffer saline with 0.1% Tween-20 for over 1 h at room temperature and then reacted them with different primary antibodies overnight at 4 °C. We incubated HRP-conjugated specific secondary antibodies for 45 min at room temperature, developing the blots using enhanced chemiluminescence (AbClon, Seoul, Korea). We quantified band intensities using the National Institutes of Health’s Image J software.

### Monocyte adhesion assay

We adhered the monocytes for 24 h to the HUVECs with human leukemic monocyte THP-1, treating the HUVECs cultured at a concentration of 2 × 10^4^ cells/well in 24-well culture plates that contained AGEs with or without PM and NAC; the THP-1 cells were labeled with 100 μM BCECF-AM for 30 min at 37 °C in a CO_2_ incubator. We co-cultured the treated HUVECs with FBS-free reagents prior to labeling the THP-1 (4 × 10^4^ cells/well) for 1 h at 37 °C. After we twice gently removed the non-adhered THP-1 cells, we lysed the cells in 0.1% SDS in 50 mM Tris–HCl, pH 7.4, and we detected the fluorescence using the fluorescence spectrophotometer with excitation at 485 nm and emission at 535 nm.

To observe the monocytes’ adhesion to the endothelial cells, we seeded the HUVECs on 12-well culture plates and treated them for 24 h with or without 10 μM PM and 1000 μM NAC that contained 100 μg/mL glycer-AGEs. After the treatments, we co-cultured the BCECF-AM-labeled THP-1 cells for 1 h. We washed the free THP-1 cells with PBS and could visualize the adhered THP-1 cells by confocal laser microscopy (Carl Zeiss, Oberkochen, Germany).

### Immunofluorescence staining

To determine the importance of NF-κB p65 nuclear translocation, we seeded the HUVECs (1 × 10^5^ cells/well) on 12-well culture plates and treated them with or without 10 μM PM and 1000 μM NAC in presence of 100 μg/mL glycer-AGEs for 4 h. We fixed the cells in 3.7% paraformaldehyde in PBS for 20 min and permeabilized them with 0.1% Triton X-100 in PBS for 15 min at room temperature. PBS washing was conducted three times in each step, each time blocked with 1% BSA in PBS for 1 h and then incubated with anti-NF-κB (p65) primary antibody in 1% BSA overnight at 4 °C. Anti-rabbit Alexa 488 fluorescence was incubated for 2 h at room temperature, and the nuclei were stained with 4′,6′-diamidino-2-phenylindole (DAPI, 500 ng/mL) for 10 min. Stained cells were washed with 1% BSA and visualized with confocal laser microscopy (Carl Zeiss, Oberkochen, Germany).

### Preparing the RNA and quantitative real-time reverse transcription-polymerase chain reaction

We harvested the HUVECs (1 × 10^6^ cells/dish) in 60-mm dishes and co-cultured the THP-1 (2 × 10^6^ cells/dish) for 1 h on the HUVECs. We co-treated10 μM PM or 1000 μM NAC that contained 100 μg/mL of glycer-AGEs in M199 medium for 6 h. We extracted total RNA using TRIzol Reagent (TAKARA Korea Biomedical Co, Seoul, Korea) and generated cDNA using the LeGene Premium Express 1st Strand cDNA Synthesis System (Legene Biosciences, SanDiego, CA, USA). We performed quantitative real-time PCR (qRT-PCR) with HiPi Real-timePCR 2X Master MixSYBR green (Elpis Biotech, Seoul, Korea) and analyzed the results using the iQ5 thermal cycler (Bio-rad, Foster City, CA, USA). The specific human mRNA primers we used in this study were as follows: RAGE (286 bp), forward primers, 5′-GGAATGGAAAGGAGACCAAG-3′, reverse primers, 5′-CCCTTCTCATTAGGCACCAG-3′; ICAM (409 bp), forward primers, 5′-TGAAGGCCACCCCAGAGGACAAC-3′, reverse primers, 5′-CCCATTATGACTGCGGCTGCTGCTACC-3′; VCAM(660 bp), forward primers, 5′-GGAACCTTGCAGCTTACAGTGACAGAGCTCCC-3′, reverse primers, 5′-CAAGTCTACATATCACCCAAG-3′; TNF-α(600 bp), forward primers, 5′-CCCAGGGACCTCTCTCTAATCA-3′, reverse primers, 5′- GCTACAGGCTTGTCACTCGG-3′; IL-6(550 bp), forward primers, 5′-GGTACATCCTCGACGGCATCT-3′, reverse primers, 5′-GTGCCTCTTTGCTGCTTTCAC-3′; MCP-1(161 bp), forward primers, 5′-TCGCGAGCTATAGAAGAATCA-3′, reverse primers, 5′-TGTTCAAGTCTTCGGAGTTTG −3′; GAPDH(550 bp), forward primers, 5′- GAAGGTGAAGGTCGGAGT-3′, reverse primers, 5′- GAAGATGGTGATGGGATTTC-3′. We analyzed the amplified genes in 1.0–1.5% agarose gels under UV light and normalized the mRNA expression levels with GAPDH expression.

### Statistical analysis

All data are quantified as mean ± standard deviation (SD) for the triplicate experiments. We used SAS version 9.3 (SAS Institute, Cary, NC, USA) to analyze the statistical differences, defining significance using Duncan’s multiple range test for *p < 0.05* for all tests.

## Results

### PM cytotoxicity and radical scavenging activity

**Fig. 1:**
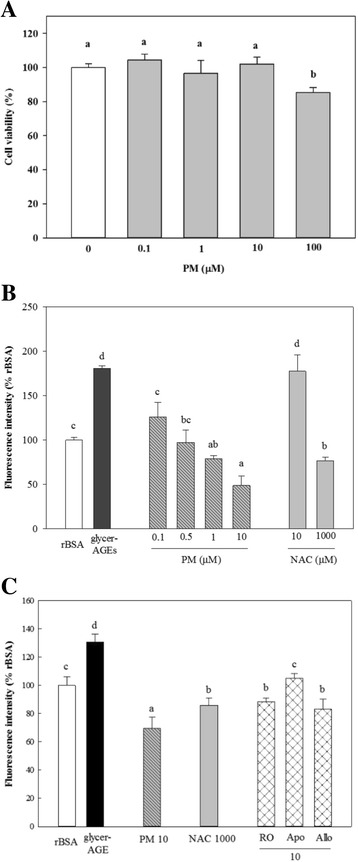
Cytotoxicity of plantamajoside (PM) and PM’s prevention of glyceraldehyde-induced advanced glycation end-products (glycer-AGEs) mediates intracellular ROS generation in HUVECs. The cytotoxicity of PM on the HUVECs was determined by MTT assay. Cells were treated with various concentrations of PM for 24 h. Intracellular ROS was measured using fluorescent DCF-DA assay. HUVECs were co-treated with various concentrations of PM (μM) and NAC (μM) including with glycer-AGEs (100 μg/mL) for 3 h. rBSA was used as the control for the sample-treated groups. **a** PM was administered for 24 h. **b** The various concentrations of PM and NAC were treated with glycer-AGEs for 3 h. **c** The different inhibitors in the cellular systems were pre-treated for 1 h, and then the samples were treated with the same methods in Fig. 1b, specifically, rotenone; mitochondrial electron transport chain inhibitors, Apocynin; NAD(P)H oxidase inhibitors, Allopurinol; xanthine oxidase inhibitors. The results were analyzed with Duncan’s multiple range test as means ± SD for triplicate experiments. Significant differences were indicated by *p < 0.05*

We incubated the HUVECs with differing concentrations of PM for 24 h (Fig. 1a) and treated them with up to 10 μMPM and 100 μg/mLof glycer-AGEs for 3 h (Fig. 1b). We checked the PM cytotoxicity at 100 μM PM for 24 h and then treated with the PM only up to 10 μM PM after all experiments; NAC was not cytotoxic up to 1 mM for 24 h (Additional file 2: Figure S2). The intracellular ROS increased significantly (*p* < 0.05) with the glycer-AGEs, whereas PM suppressed ROS formation in a concentration-dependent manner. 10 μM PM (49 ± 10% of rBSA) reduced glycer-AGE-mediated ROS generation by significantly (*p* < 0.05) more than 1000 μM NAC (77 ± 4% of rBSA). In addition, we pre-treated cells with specific inhibitors to investigate the main sources of ROS by glycer-AGEs for 1 h prior to treatment with the glycer-AGEs (Fig. 1c). The ROS formation by glycer-AGEs was significantly (*p* < 0.05) decreased with 10 μM rotenone (a mitochondrial electron transport inhibitor), apocynin (an NAD(P)H oxidase inhibitor), and allopurinol (a xanthine oxidase inhibitor). AGE was confirmed as inducing cellular stress compared with rBSA. After all of the experiments, we focused on the glycer-AGE-induced ROS-mediated signals against the control (CON), treated with free media, without the rBSA groups.

### Effect of PM in HUVECs treated with glycer-AGEs

**Fig. 2:**
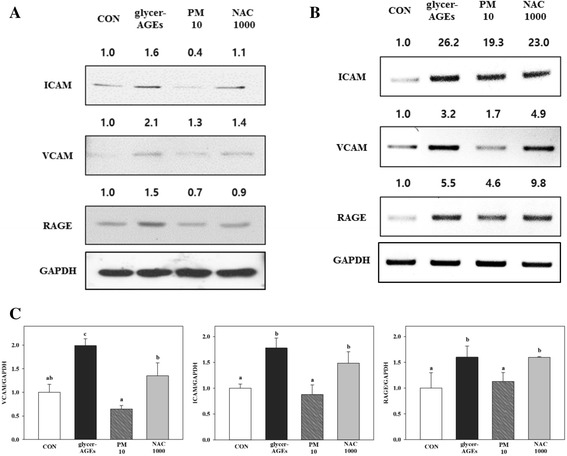
PM regulation of receptor for AGEs (RAGE) and adhesion molecule expression in HUVECs treated with glycer-AGEs. HUVECs were co-treated with various concentrations of PM (10 μM) and NAC (1000 μM) including with glycer-AGEs (100 μg/mL) for 24 h, and then the cell lysates were obtained and analyzed with Western blotting or qRT-PCR. **a** Total cell lysates were obtained by RIPA that contained protease inhibitors and separated by 10% SDS-PAGE with GAPDH as a control. The relative changes in protein bands were measured by Image J software. **b** mRNA was collected with TRIzol reagent and analyzed by 1.2-2% agarose gel with GAPDH as a control. The relative changes in mRNA bands were measured by Image J software. **c** Quantitative real-time PCR (qRT-PCR) analyzed by HiPi Real-timePCR 2X Master Mix SYBR green (Elpis Biotech, Seoul, Korea) and analyzed using iQ5 thermal cycler (Bio-rad, CA, USA) with GAPDH as a control. Significance differences were analyzed with Duncan’s multiple range test as means ± SD at *p < 0.05*

We examined the effects of PM on the RAGE protein and mRNA expression levels and the adhesion molecules in the HUVECs treated with glycer-AGEs (Fig. 2); after 24 h treatments with the glycer-AGEs in the absence of PM, the RAGE protein and mRNA levels increased notably, 1.5 and 5.5 times, respectively. In contrast, the co-treatment with 10 μM PM and 100 μg/mLof glycer-AGEs reduced protein expression (Fig. 2a), and mRNA expression was also suppressed with PM (Fig. 2b). Based on the qRT-PCR analysis, the PM strongly suppressed glycer-AGE-induced RAGE mRNA levels and adhesion molecule expression in the HUVECs (Fig. 2c). In the same manner, protein and mRNA expression of the adhesion molecules in the cells were elevated with glycer-AGE treatment, whereas co-treatment with PM suppressed their stimulation.

### Effects of PM on monocyte adhesion to HUVECs

**Fig. 3:**
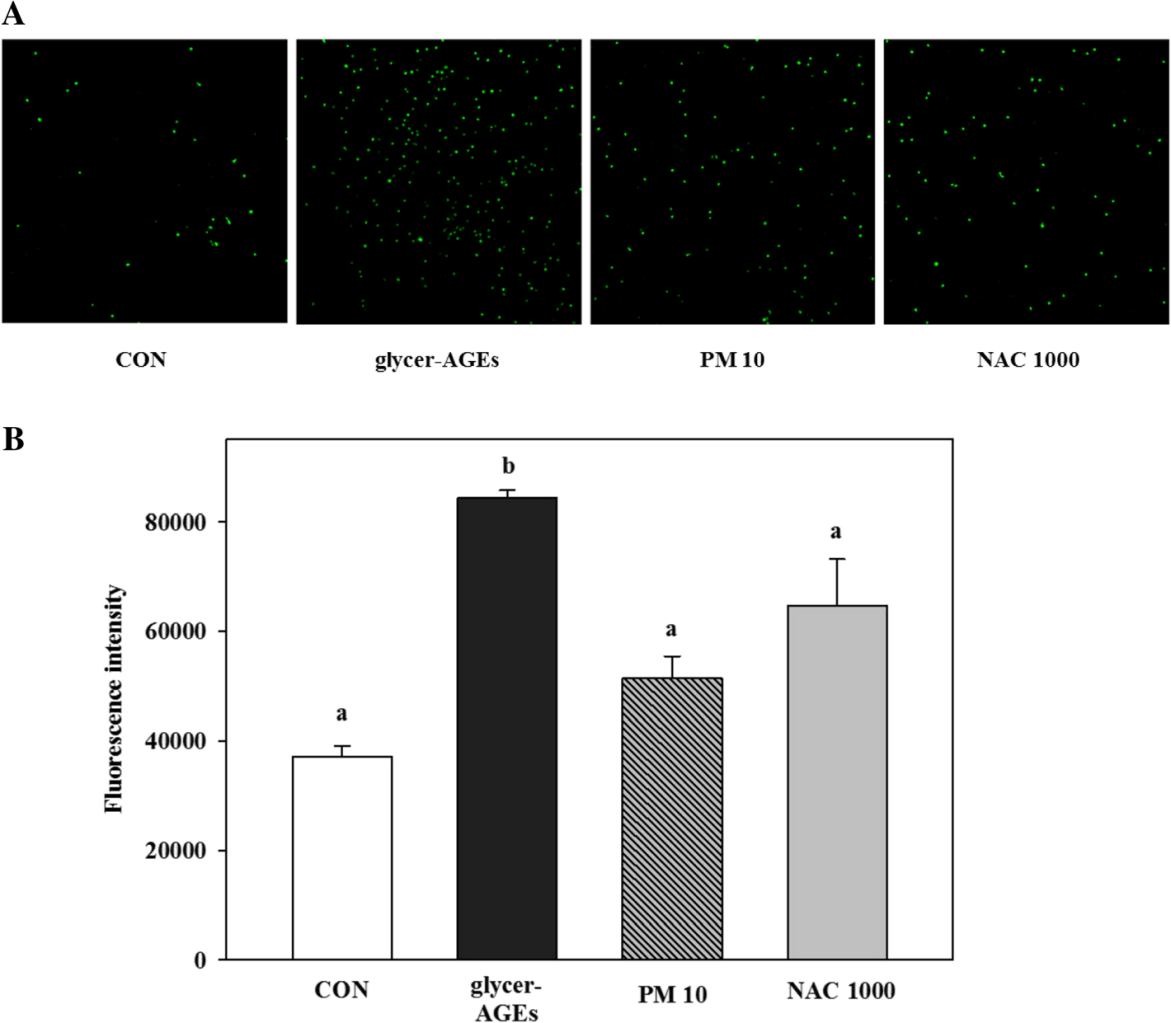
PM regulation of glycer-AGE-mediated monocyte and THP-1 adhesion to HUVECs. HUVECs were co-treated with PM (10 μM) and NAC (1000 μM) including glycer-AGEs (100 μg/ml) for 24 h, and then THP-1 cells were co-cultured on the HUVECs for 1 h in a dark CO_2_ incubator. Before the co-culture, the THP-1 cells were pre-labeled by BCECF-AM (100 μM) for 30 min. After the co-culture periods, the unattached monocytes were washed twice with PBS. **a** BCECF-AM-labeled monocytes were detected using confocal microscopy (100× magnification). **b** After the co-culture periods, cells lysis by 0.1% SDS in 50 mM Tris-buffer (pH 7.0) was detected using a fluorescence multi-plate reader in excitation 485 nm, emission 535 nm. The fluorescence was quantified and analyzed with Duncan’s multiple range test as means ± SD. Significant differences were indicated by *p < 0.05*

To evaluate the influence of PM on the monocytes’ interaction with the endothelial cells, we used confocal microscopy to confirm the THP-1 adhesion to endothelial cells treated with glycer-AGEs (Fig. 3). We observed remarkably increased monocyte adhesion to the endothelial cells in the group treated with glycer-AGEs compared with the untreated control group, whereas PM treatment with the glycer-AGEs suppressed the adhesions (Fig. 3a). The fluorescent intensity of the individual monocyte adhesions also confirmed that cells treated with both PM and glycer-AGEs showed suppressed fluorescent intensity compared with cells treated with glycer-AGEs alone (Fig. 3b).

### Effects of PM in HUVECs co-cultured with monocytes treated with glycer-AGEs

**Fig. 4:**
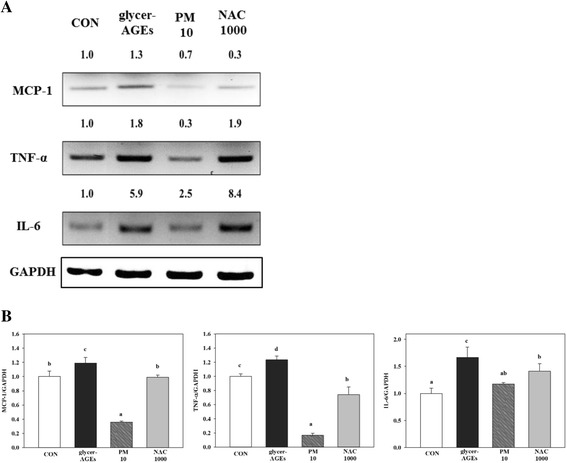
PM regulation of glycer-AGE-mediated pro-inflammatory cytokines in HUVECs with monocytes and THP-1. After the HUVECs and THP-1 cells were co-cultured, they were co-treated with PM (10 μM) and NAC (1000 μM) including glycer-AGE (100 μg/mL) for 6 h. After the treatments, the pro-inflammatory cytokines were measured using qRT-PCR. **a** mRNA was collected with TRIzol reagent and analyzed by 1.2–2% agarose gel with GAPDH as a control. The relative changes in mRNA bands were measured by Image J software. **b** qRT-PCR was analyzed by HiPi Real-timePCR 2X Master Mix SYBR green (Elpis Biotech, Seoul, Korea) using iQ5 thermal cycler (Bio-rad, CA, USA) with GAPDH as a control. Significance differences were analyzed with Duncan’s multiple range test as means ± SD and indicated by *p < 0.05*

It is known that pro-inflammatory cytokines mediate the adhesion of monocytes to endothelia by activating the immune response [34–36], and we measured cytokine mRNA expression with glycer-AGE treatment in endothelia that were co-cultured with monocytes (Fig. 4a). Based on our qRT-PCR analysis (Fig. 4b), the TNF-α, IL-6 and MCP-1 mRNA levels in the HUVECs and THP-1 co-cultures were significantly (*p* < 0.05) increased with glycer-AGE treatment, whereas PM treatment significantly (*p* < 0.05) reduced the mRNA levels of these pro-inflammatory cytokines. PM also differed significantly (*p* < 0.05) from CON.

### Effects of PM on NF-κB in HUVEC cells treated with glycer-AGEs

**Fig. 5:**
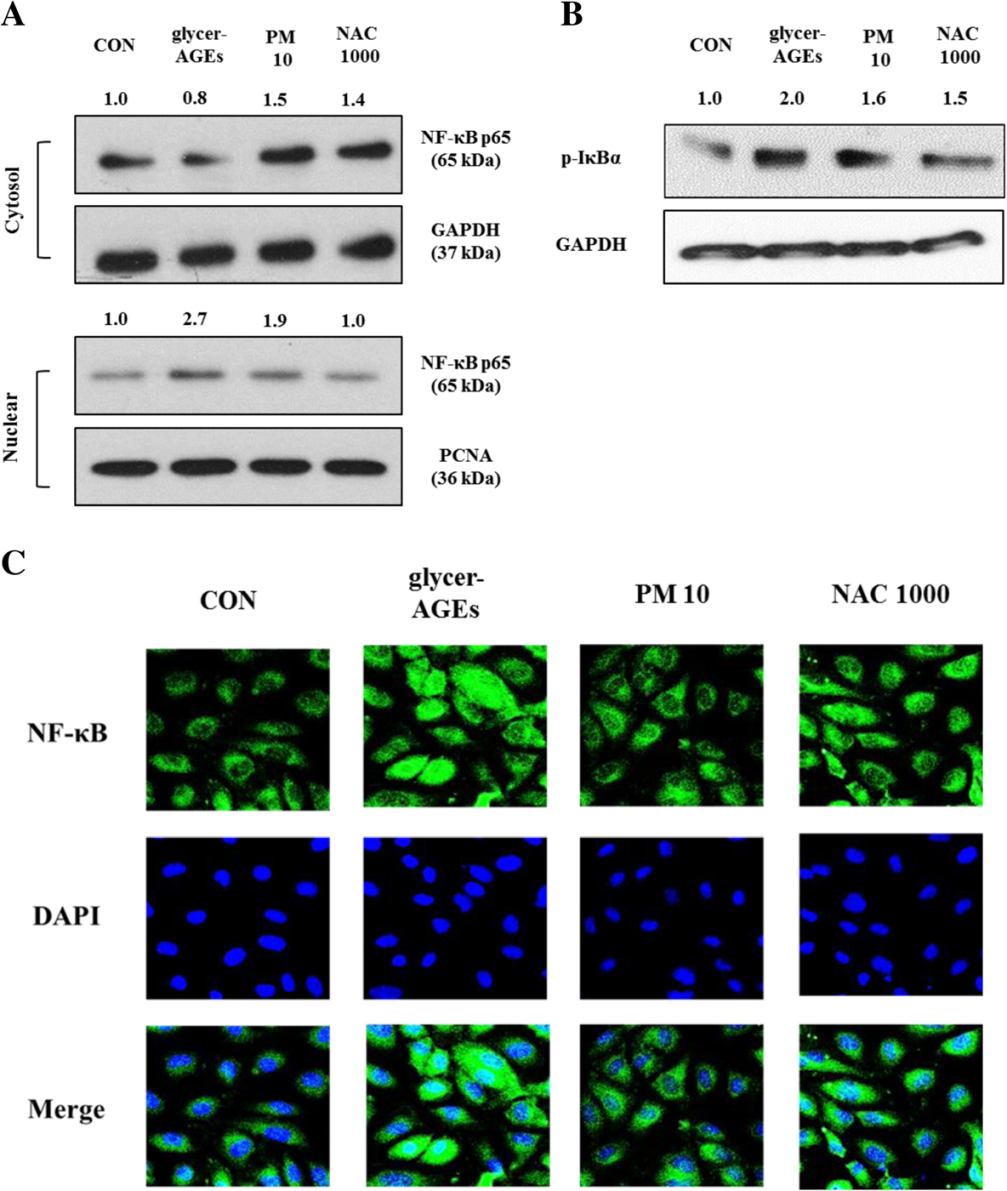
PM regulation of glycer-AGE-mediated NF-κB activation. HUVECs were co-treated with PM (10 μM) and NAC (1000 μM) including glycer-AGEs (100 μg/mL) for 4 h. NF-κB activation was detected by Western blotting or immunofluorescence staining, and the relative changes in mRNA bands were measured by Image J software. **a** After co-treatments, cells were collected with PBS for nucleic fraction and analyzed with 10% SDS-PAGE with GAPDH and PCNA as a control against the p65 antibody. **b** Total cell lysates were obtained by RIPA that contained protease inhibitors, and a phosphatase inhibitor cocktail, and then separated by 10% SDS-PAGE with GAPDH as a control. **c** Immunofluorescence staining shows the translocation of NF-κB to the nucleus. After co-treatments, the cells were fixed, permeabilized, and then incubated with anti-p65 antibody overnight. The cell nuclei were stained with DAPI (500 ng/mL) for 5 min and visualized using confocal microscopy

Both pro-inflammatory cytokines and adhesion molecule expression are known to be regulated by NF-κB [37, 38]. NF-κB is a heterodimeric protein, and its activation is progressed by IκB kinase, which phosphorylates IκB in cytoplasm. Phosphorylated IκB leads to its degradation from the NF-κB-IκB complex, freeing dimers of p65 and p50 to translocate to the nucleus, binding them to NF-κB DNA response elements and inducing the transcription of the target genes [37]. We elucidated that the glycer-AGE treatment stimulated p65 subunit of NF-κB in the cytosol and the nuclear fraction (Fig. 5a) and in the total phosphorylation of IκB (Fig. 5b). As we expected, co-treatment with PM and glycer-AGEs attenuated the p65 level in the nuclei and the IκB phosphorylation. Confocal microscopic examination for p65 nuclear trans-localization also confirmed that glycer-AGE-activated NF-κB translocation was inhibited by PM treatment (Fig. 5c).

### Effects of PM on MAPK signaling in HUVECs treated with glycer-AGEs

**Fig. 6:**
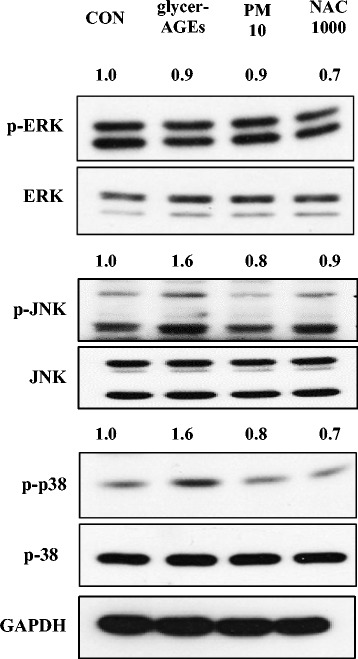
PM regulation on glycer-AGE-mediated MAPK signaling in HUVECs. The HUVECs were co-treated with various concentrations of PM (10 μM) and NAC (1000 μM) including with glycer-AGEs (100 μg/mL) for 60 min., and then the cell lysates were obtained and analyzed with Western blotting. Total cell lysates were obtained by RIPA that contained protease inhibitors and a phosphatase inhibitor cocktail and then separated by 10% SDS-PAGE with GAPDH as a control

To further elucidate which MAPK signal pathways are involved in treating HUVECs with glycer-AGEs, we treated the cells with glycer-AGEs with and without 10 μM PM for 60 min (Fig. 6). The glycer-AGEs slightly increased the phosphorylation of the JNK and p38 pathways (both by 1.6 times), whereas the phosphorylation was suppressed with co-treatment with PMs. In contrast, the ERK pathway was not affected by any treatment with glycer-AGEs or PM.

### Effects of PM on signaling pathways in HUVECs treated with glycer-AGEs

**Fig. 7:**
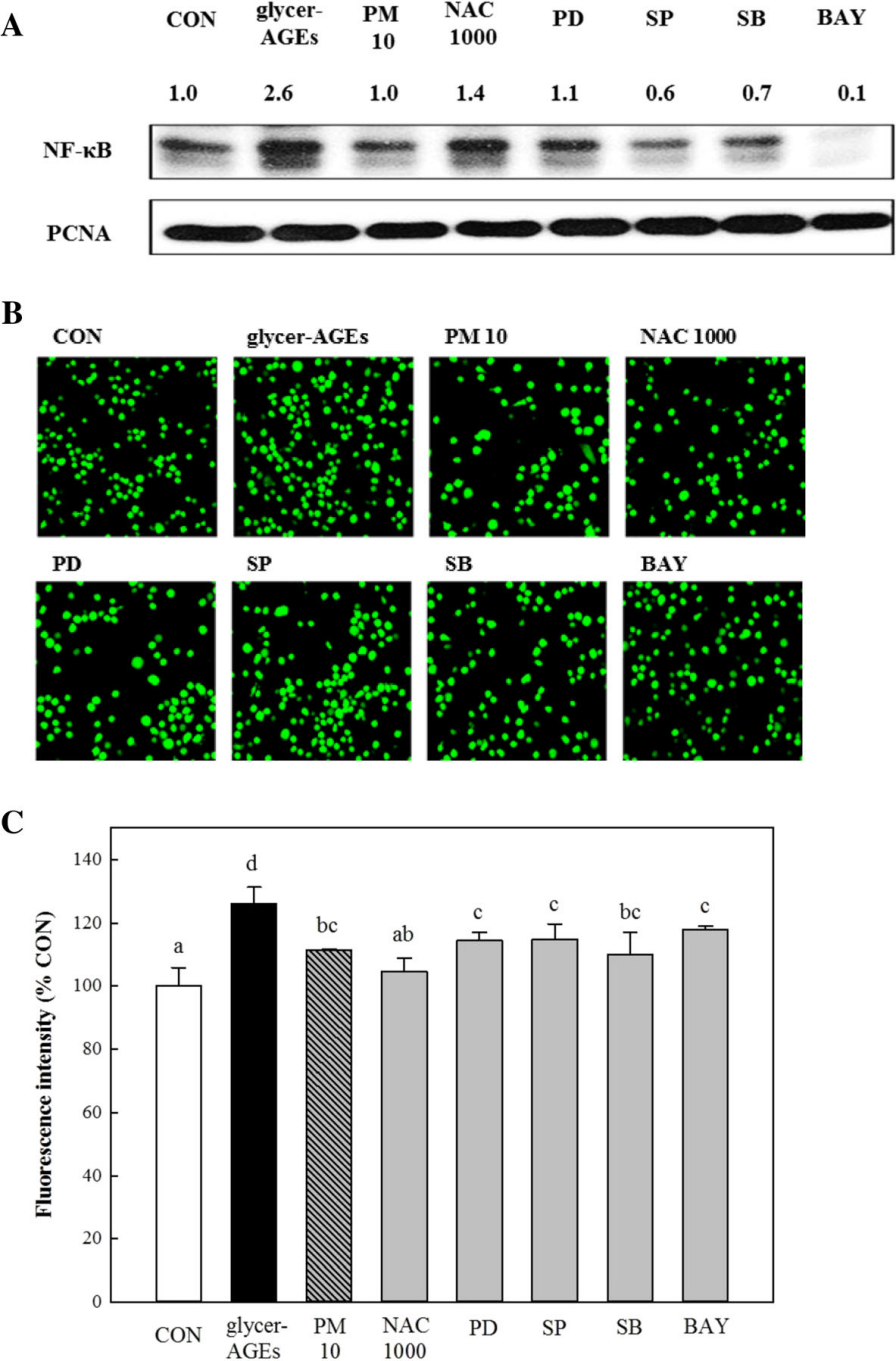
PM regulates glycer-AGE-mediated cellular signaling for attracting endothelial dysfunction. The HUVECs were co-treated with various concentrations of PM (10 μM) and NAC (1000 μM) including with glycer-AGEs (100 μg/mL) for the indicated treatment times. The specific inhibitors (10 μM) were pre-treated before the glycer-AGE stimulation in all experiments, specifically: PD98059; the ERK inhibitor, SP600125; the JNK inhibitor, SB203580; the p38 inhibitor, BAY11-7082; and the NF-κB inhibitor. Relative expression of control was analyzed using Image J. **a** The HUVECs were co-treated with PM (10 μM) and NAC (1000 μM) including glycer-AGEs (100 μg/mL) for 4 h, and then the cells were collected with PBS for nucleic fraction and analyzed with 10% SDS-PAGE with PCNA as a control. Relative quantification of the bands was analyzed using Image J software. **b** The HUVECs were co-treated with PM (10 μM) and NAC (1000 μM) including glycer-AGEs (100 μg/mL) for 24 h, and then the THP-1 cells were co-cultured on the HUVECs for 1 h in a dark CO_2_ incubator. Before the co-culture, the THP-1 cells were pre-labeled by BCECF-AM (100 μM) for 30 min. After the co-culture periods, the un-attached monocytes were washed twice with PBS. BCECF-AM-labeled monocytes were detected using confocal microscopy (100× magnification). **c** The cells were treated with the same method in Fig. 7b. After the co-culture periods, cell lysis by 0.1% SDS in 50 mM Tris-buffer (pH 7.0) was detected using a fluorescence multi-plate reader in excitation 485 nm and emission 535 nm. The fluorescence was quantified and analyzed with Duncan’s multiple range test as means ± SD at *p < 0.05*

Next, we treated cells with an NF-κB (BAY11-7082) and MAPK, ERK (PD98059), JNK (SP600125), and p38 (SB203580) inhibitors and observed that NF-κB translocation into the nuclei decreased with these inhibitors without PD98059, which is effective in ERK signaling to NF-κB translocation (Fig. 7a). In addition, the increased adhesion of monocytes to endothelial cells treated with glycer-AGEs was reduced with treatment with PM, and all specific inhibitors with glycer-AGEs suppressed these adhesions (Fig. 7b). The fluorescent intensity of the individual monocyte adhesions also confirmed these observations (Fig. 7c).

## Discussion

Studies have reported that *P. asiatica* has bioactive activities as a number of phytochemical agents [29, 30]. In particular, PM purified from *P. asiatica* is a phenylpropanoid glycoside that contains caffeic acid derivatives [31, 33]; nevertheless, the molecular mechanism of how PM modulates endothelial dysfunction remains uncertain. Our groups have reported that glycolaldehyde-derived AGEs stimulated intracellular ROS production and pro-inflammatory mediators including TNF-α and IL-1β via the AGE-RAGE axis [39]. One recent study showed that co-treatment with PM and glycer-AGEs in keratinocytes and fibroblasts inhibits UVB-irradiation- and AGE-induced RAGE overexpression and proinflammatory cytokine expression via attenuating MAPK activation by ROS [40]. Given that PM is absorbed very rapidly into the blood in rats after oral administration of *P. asiatica* extract [41], in the present study, we co-treated PM with glycer-AGEs in HUVECs to inhibit intracellular ROS production via AGE-RAGE interaction. In the study, we confirmed that PM suppressed monocyte adhesion by glycer-AGEs and inflammation cascades in endothelia, and we showed preventive effects on NF-κB translocation and phosphorylation of JNK and p38 in HUVECs by glycer-AGEs.

It has been reported that AGEs accelerate cellular inflammation [9–12]. In particular, intracellular ROS are critical in inducing inflammation in cells and play a critical role in endothelium activation related with vascular diseases including atherosclerosis; the chronic oxidative stress could activate a number of different signal pathways including MAPK and NF-κB [42]. In the present study, PM showed high inhibitory activity against glycer-AGE-mediated intracellular ROS generation with 10 μM of the compound (49 ± 10% of rBSA), whereas NAC inhibited the comparable ROS generation (77 ± 4% of rBSA) at the 1000 micromolar level. In addition, our study showed that glycer-AGEs increased intracellular ROS via not only NAD(P)H oxidase with apocynin and mitochondrial electron transportation with rotenone but also from xanthine oxidase with allopurinol in HUVECs. This suggested that glycer-AGEs are multiple sources of generating ROS in cellular systems. NAC has been reported as a major antioxidant reagent in a wide variety of experiments.

Monocytes adhere to endothelia with the expression of adhesion molecules including VCAM and ICAM as well as inflammatory cytokines both in vitro and in vivo [19, 20, 43, 44]. Through the adhesion molecules, circulating leukocytes adhere to endothelial cells and invade endothelial barriers under various stimuli, and these leukocytes secrete multiple inflammatory cytokines, resulting in chronic inflammatory diseases [21], and endothelial dysfunction with adhered monocytes is a key to the sclerotic inflammation that is a feature of diabetes complications [45]. Other authors have demonstrated that phytochemicals such as sulforaphane in cruciferous vegetables [46], acteosides in flowers [47], green tea derivatives [48] and curcumin [49] have potentially preventive effects on endothelial dysfunction. Our data confirmed that PM inhibits the expression of adhesion molecules and blocks monocyte adhesion to endothelial cells as well.

In diabetes, RAGE activation induces a variety of inflammatory cytokines resulting from the NF-κB activation, including TNF-α, IL-6 and MCP-1, in the vascular system [50–52], and these relevant inflammatory cytokines contribute to the development of early atherosclerosis [53]; in addition, with adhesion molecules, MCP-1 contributes to the transmigration and infiltration of monocytes to endothelia [54, 55]. Our study confirmed that treatment with PM significantly reduces the glycer-AGE-induced mRNA levels of TNF-α, IL-6, and MCP-1 under THP-1 co-cultured conditions. For chronic initiation states of inflammatory cytokine-related sclerotic diseases, leukocytes with HUVECs are more critical for expressing the pro-inflammatory cytokines, and not only in HUVECs; NF-κB activation was closely involved in the inflammation response in the cellular system with MAP kinase pathways. There have been diverse NF-κB hypotheses regarding the connection(s) between MAPK signaling and NF-κB that systematized cell responses [56–59]. Based on our results, glycer-AGEs increased nuclear translocation of NF-κB and activated the phosphorylation of JNK and p38 MAPK in HUVECs, but PM inhibited this glycer-AGE-triggered JNK and p38 activity. In addition, treatments with PM, as well as JNK, p38 and NF-κB inhibitors, significantly blocked monocytes’ adhesion to endothelial monolayers against glycer-AGEs, suggesting that PM may be a pivotal regulator in the vascular inflammation that induces endothelial dysfunction via the MAPK/NF-κB pathways.

## Conclusions

In this study, we demonstrated that PM inhibits inflammation-induced monocyte adhesion by suppressing adhesion molecules via down-regulation of the NF-κB pathway. These approaches might contribute to elucidating the mechanism of PM’s preventive action. Therefore, we may have provided the first approach to using PM as a potential natural compound to protect endothelial cells against inflammatory cellular dysfunction.

## Additional files

Additional file 1:
**Figure S1.** Advanced glycation end-product formation. Bovine serum albumin and glyceraldehyde were mixed at 37 °C in the dark for 7 days. The fluorescence was measured using fluorescence intensity set at excitation 370 nm and emission 440 nm. (DOCX 71 kb)
Additional file 2:
**Figure S2.** Cytotoxicity of *N*-acetylcysteine (NAC). The NAC cytotoxicity in the HUVECs was determined by MTT assay. Cells were treated with various concentrations of NAC for 24 h. Results were analyzed with Duncan’s multiple range test as means ± SD for triplicate experiments. Significant differences were indicated by *p < 0.05*.(DOCX 69 kb)

## Abbreviations

μM: Micromolar
AGEs: Advanced glycation end-products
Allo: Allopurinol
Apo: Apocynin
BCECF-AM: 2′,7′-bis-(2-carboxyethyl)-5-(and-6)-carboxyfluorescein, acetoxymethyl ester
BSA: Bovine serum albumin
DCF-DA: Dichlorodihydrofluorescein diacetate
EGM-2: Endothelium growth media-2
ERK: Extracellular signal-regulated kinase
Glycer: Glyceraldehyde
HUVECs: Human umbilical vein endothelial cells
ICAM: Intercellular adhesion molecule
IL-6: Interleukin-6
JNK: c-Jun N-terminal kinase
MAPK: Mitogen-activated protein kinase
MCP-1: Monocyte chemoattractant protein-1
NAC: *N*-acetylcysteine
NF-κB: Nuclear factor-kappa B
PM: Plantamajoside
PMSF: Phenylmethane sulfonyl fluoride
RAGE: Receptor for advanced glycation end-products
rBSA: Reducted bovine serum albumin
RO: Rotenone
ROS: Reactive oxygen species
SDS-PAGE: Sodium dodecyl sulfate-polyacrylamide gel electrophoresis
TNF-α: Tumor necrosis factor-alpha
VCAM: Vascular cellular adhesion molecule
