# Supplementary material for: Plantamajoside from Plantago asiatica modulates human umbilical vein endothelial cell dysfunction by glyceraldehyde-induced AGEs via MAPK/NF-κB
Source: BMC Complement Altern Med. 2017 Jan 21;17:66. doi: 10.1186/s12906-017-1570-1 (PMC5251346; doi:10.1186/s12906-017-1570-1)

**Figure S1. Advanced glycation end-product formation.** Bovine serum albumin and glyceraldehyde were mixed at 37°C in the dark for 7 days. The fluorescence was measured using fluorescence intensity set at excitation 370 nm and emission 440 nm.

**Figure S1**


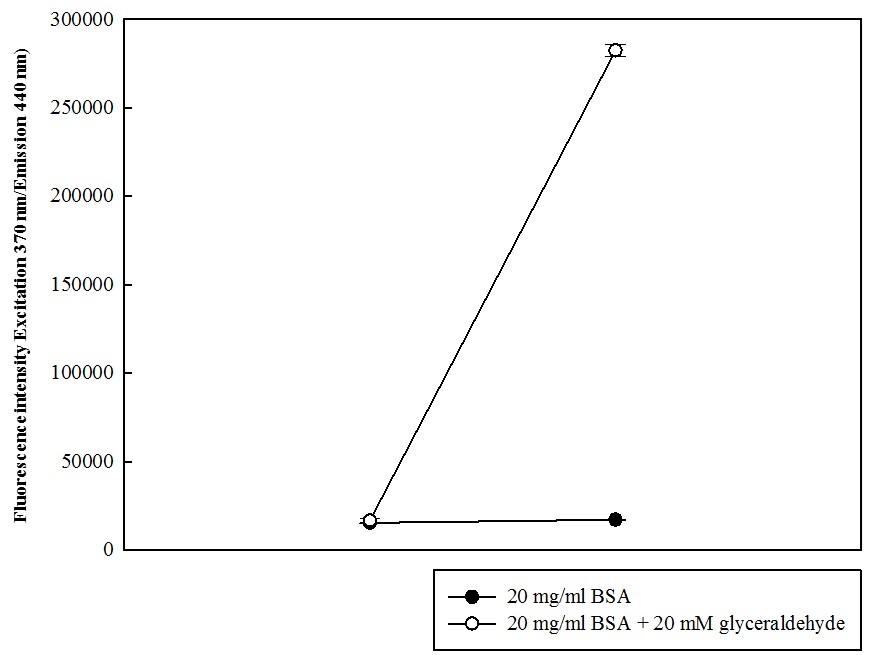

Supplement: Additional file 1: — Figure S1. Advanced glycation end-product formation. Bovine serum albumin and glyceraldehyde were mixed at 37 °C in the dark for 7 days. The fluorescence was measured using fluorescence intensity set at excitation 370 nm and emission 440 nm. (DOCX 71 kb) [file 12906_2017_1570_MOESM1_ESM.docx]
