# Supplementary material for: Plantamajoside from Plantago asiatica modulates human umbilical vein endothelial cell dysfunction by glyceraldehyde-induced AGEs via MAPK/NF-κB
Source: BMC Complement Altern Med. 2017 Jan 21;17:66. doi: 10.1186/s12906-017-1570-1 (PMC5251346; doi:10.1186/s12906-017-1570-1)

**Figure S2. Cytotoxicity of *N*-acetylcysteine (NAC).** The NAC cytotoxicity in the HUVECs was determined by MTT assay. Cells were treated with various concentrations of NAC for 24 h. Results were analyzed with Duncan’s multiple range test as means ± SD for triplicate experiments. Significant differences were indicated by *p<0.05*.

**Figure S2**


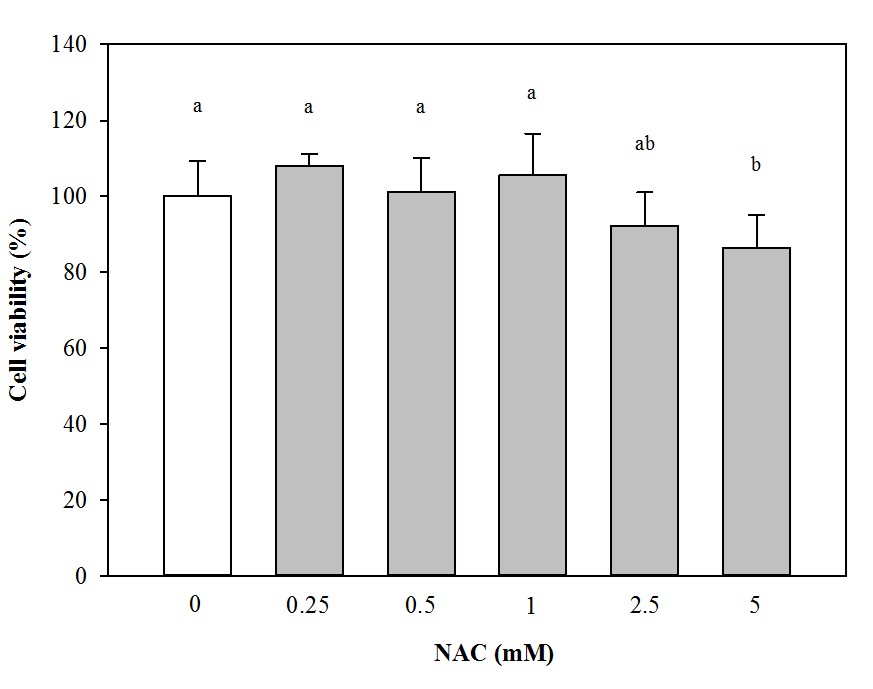

Supplement: Additional file 2: — Figure S2. Cytotoxicity of N-acetylcysteine (NAC). The NAC cytotoxicity in the HUVECs was determined by MTT assay. Cells were treated with various concentrations of NAC for 24 h. Results were analyzed with Duncan’s multiple range test as means ± SD for triplicate experiments. Significant differences were indicated by p < 0.05.(DOCX 69 kb) [file 12906_2017_1570_MOESM2_ESM.docx]
